# Obesity-related Parameters as Potential Contributors to Increased Headache Frequency in Migraineurs

**DOI:** 10.4314/ejhs.v35i4.9

**Published:** 2025-07

**Authors:** Saima Nazish

**Affiliations:** 1 Department of Neurology, College of medicine, Imam Abdulrahman Bin Faisal University, Dammam, Saudi Arabia

**Keywords:** Migraine, Obesity, Headache Frequency, BMI, Waist Circumference, WHtR

## Abstract

**Background:**

Migraine and obesity often coexist, and both generalized and central obesity may influence the frequency and severity of migraine attacks. This study aimed to investigate the association between obesity-related anthropometric measures and headache burden in individuals with migraine.

**Methods:**

This observational study was conducted at King Fahad Hospital of the University, Saudi Arabia, and included 186 patients diagnosed with migraine. Anthropometric data were collected at baseline and again after a minimum follow-up period of 12 months. Headache frequency and severity were evaluated, alongside clinical variables such as dietary habits and medication adherence.

**Results:**

Elevated body mass index (BMI), waist circumference (WC), and waist-to-height ratio (WHtR) were observed in 43.1%, 67.2%, and 41.4% of participants, respectively. At follow-up, 51.6% of patients reported an increase in headache frequency, with significantly higher BMI values among this subgroup (p = 0.018). Increased headache frequency was significantly associated with elevated BMI (p = 0.029), high WHtR (p = 0.023), and follow-up increases in both body weight and WC (p < 0.01). However, in multivariate logistic regression analysis, only follow-up increases in weight and WC remained marginally associated with increased headache frequency (OR: 1.94, 95% CI: 0.97–3.89; p = 0.05). Baseline values for BMI, WC, and WHtR were not independent predictors.

**Conclusion:**

While obesity-related measures were prevalent among migraine patients, they were not independent predictors of increased headache frequency when behavioral factors were considered. These findings underscore the need for a comprehensive approach to migraine management, emphasizing lifestyle modifications.

## Introduction

Migraine is a primary headache disorder characterized by recurrent, unilateral, pulsatile headaches lasting 4–72 hours, often accompanied by other neurological symptoms (1). Its etiology is multifactorial, involving genetic and environmental influences, as well as factors like dietary imbalance, physical inactivity, inflammation, and hypothalamic dysfunction (2). Chronic migraine has been linked to obesity in population-based studies, and obese individuals with episodic migraine are at risk of progressing to chronic migraine (3-4).

Obesity may exacerbate migraine frequency and severity, potentially through the release of inflammatory mediators—particularly from visceral adipose tissue—that influence migraine pathophysiology (5). Elevated levels of proinflammatory cytokines such as IL-6, CRP, and TNF-α have been detected in both episodic and chronic migraine sufferers compared to controls.(6) Generalized obesity is especially prevalent among reproductive-age female migraineurs, (7) while central (abdominal) obesity appears more closely associated with migraine in individuals under 50 (8-9) This may be due to adipokine-mediated effects on the hypothalamus—a key regulator of appetite, weight, and migraine initiation (10).

Our center previously found no significant links between body mass index (BMI) categories and migraine characteristics, although abdominal obesity and its relationship with headache frequency were not examined (11). This study aims to evaluate how generalized and abdominal obesity—reflected by BMI, waist circumference (WC), and waist-to-height ratio (WHtR)—relate to headache frequency, severity, and comorbidities. We hypothesize that both obesity types contribute to increased migraine burden in our population, warranting careful consideration in patient management.

## Materials and Methods

This observational study was conducted in the Neurology Department at King Fahd Hospital of the University from September 2021 to March 2024 (IRB-2021-01-281). Using a prevalence estimate of 45% obesity among migraineurs, a 95% confidence interval, and 7% margin of error, the required sample size was 180, but 186 patients were enrolled. Inclusion criteria were adults of either sex meeting IHS migraine criteria—with or without aura—while exclusions included other headache types, pregnancy, incomplete data, loss to follow-up, or refusal to participate.

Following informed consent, electronic records were reviewed for demographics, comorbidities, migraine duration, headache frequency and severity, and preventive medication use. At baseline, trained nursing staff measured blood pressure (after a 5-minute rest), height, weight, BMI, and WC (midpoint between the iliac crest and lowest rib) using standardized procedures. WHtR was calculated as WC divided by height. Follow-up assessments were conducted after at least 12 months to record changes in anthropometric measures, headache patterns, and medication adherence.

### The following operational definitions are used

**Migraine**: IHS criteria—≥5 attacks lasting 4–72 h with ≥2 of unilateral location, pulsating quality, moderate/severe intensity, or aggravated by routine activity, plus nausea, photophobia, or phonophobia.(12)

**Episodic migraine**: <15 headache days/month; Chronic migraine: ≥15 days/month, ≥8 of which are migrainous, for ≥3 months.(12)

**High-frequency episodic**: 8–14 migraine days/month.

**Pain intensity**: 1–3 = mild, 4–6 = moderate, 7–10 = severe.

**Generalized obesity**: BMI ≥ 30 kg/m^2^ (WHO).

**Abdominal obesity**: WC ≥ 92 cm (men), ≥ 87 cm (women)(13); WHtR > 0.5(14).

**Hypertension**: BP ≥ 130/85 mmHg.

**Poor dietary habits**: high intake of fried/fast foods, low fruit, vegetable, legume, and fish consumption.

**Statistical analysis**: Data analysis used SPSS v22. Categorical variables were compared using chisquare tests; continuous variables by t-tests or ANOVA. Variables with p<0.05 in univariate analysis were entered into multivariable logistic regression using backward stepwise elimination. Results are reported with adjusted odds ratios (OR) and 95% confidence intervals; p<0.05 was considered significant.

## Results

**Demographics and clinical characteristics**: Among 186 migraine patients (148 F, 38 M), mean age was 40.6 years, with a higher average observed in patients with comorbidities (p=0.021). While mean disease duration was 8.4 years (Table 1). Comorbidities (hypertension, depression, autoimmune/endocrine disorders) were present in 99(53.2%) patients and were significantly more common in those over 40 years of age (p=0.030). Most (87.1%) had episodic migraine; within this group, 23.1% had high-frequency attacks and 12.9% had chronic migraine.

**Table 1:** Comparison of clinical profile and anthropometric parameters of migraineurs with and without known comorbidities

Patient's clinical characteristics	All patients (n = 186) No (%)	Migrainers without comorbidities N=87	Migrainers with comorbidities N=99	p-value
**Clinical parameters**				
Sex				
Male	38(20.4)	19(21.8)	19(19.2)	0.655*
Female	148(79.6)	68(78.2)	80(80.8)	
Age, years				
< 40	89(47.8)	49(56.3)	40(40.4)	0.030*
≥ 40	97(52.2)	38(43.7)	59(59.6)	
Episodic Migraine	162(87.1)	72(82.8)	90(90.9)	0.098*
Chronic Migraine	24(12.9)	15(17.2)	9(9.1)	0.596*
High headache frequency (8-14)	43(23.1)	21(24.2)	22(22.2)	0.768*
Age, years (Mean±SD),	40.64±13.21	37.39±12.93	43.49±12.86	0.021†
Range	(14-72)	(14-61)	(15-72)	
Mean duration of diagnosis	8.38±7.00	7.67±7.15	9.01±6.84	0.196†
Mean frequencies of attacks	7.58±7.02	8.87±8.23	6.43±5.44	0.021†
Systolic BP (Mean±SD)	127.97±15.98	125.21±13.82	130.39±17.38	0.027†
Diastolic BP (Mean±SD)	79.48±11.27	78.41±11.13	80.42±11.37	0.226 †
**Anthropometric Parameters**				
Height (Mean±SD)	159.57±8.61	160.47±9.17	158.78±8.05	0.183†
Weight	74.66±18.94	72.53±17.32	76.54±20.15	0.151†
BMI	29.41±7.22	28.24±6.22	30.45±7.89	0.037†
Waist circumference	94.69±15.84	91.91±15.14	97.14±16.12	0.024†
Follow up waist circumference	95.11±16.23	91.63±15.85	98.16±16.02	0.006†
Follow up weight	75.25±18.96	72.50±17.04	77.67±20.26	0.064†

BP=Blood pressure; BMI=Body mass index; SD=standard deviation

* p value by Chi square test

† p value by independent t-test

**Anthropometric measures**: Mean weight was 74.7 ± 18.9 kg. Generalized obesity (BMI ≥30) was found in 43.1%, abdominal obesity by WC (67.2%) and WHtR (41.4%). While obesity measures did not significantly differ by comorbidity status, BMI was higher in those with comorbidities (p=0.037). Higher mean headache frequency were observed among those without comorbidities, p=0.021).

**Follow-up outcomes (12 months)**: Increased headache frequency and intensity were reported by 51.6%. Weight and WC increases occurred in 41.4%, more frequently in those with comorbidities (p<0.010; Table 2). Although overall changes in weight, BMI, or WC were not significant, those with worsened headache frequency had higher BMI (p=0.018; Table 3). While both obesity types had higher numeric headache values, differences over time were not statistically significant (p=0.947, 0.086; Table 4).

**Table 2:** Associations of patient's clinical and anthropometric parameters with high headache frequencies severity and change in frequency and severity of migraine attacks

Patient's clinical characteristics	Patients with high headache frequencies (8 to 14) N=43 N(p-value)	Patients with high headache severity N=47 N(p-value)	Change in frequency and severity of headaches N = 96 N(p-value)
Sex			
Male/Female	6/37(0.228)	6/40(0.137)	16/80(0.177)
Episodic Migraine	42(0.005)	39(0.601)	79(0.043)
Age less than 40	20(0.844)	27(0.087)	20(0.837)
Patients with comorbidities	22(0.750)	21(0.252)	49(0.529)
Known triggers	27(0.167)	34(0.018)	55(0.309)
Poor Dietary habits	23(0.186)	23(0.411)	57(0.049)
Preventive therapy	27(0.518)	28(0.688)	51(0.108)
Non-Compliance to medications	23(0.590)	22(0.149)	62(0.026)
High BP	11(0.193)	11(0.110)	36(0.279)
High BMI	19(0.918)	23(0.229)	**49(0.029)**
High waist	26(0.284)	31(0.952)	70(0.078)
High Waist height ratio	18(0.942)	21(0.464)	**47(0.023)**
High FU body weight and waist	21(0.249)	24(0.072)	**49(<0.01)**

* BP= Blood pressure; BMI=Body mass index, Fu= Followup

**Table 3:** Longitudinal changes in obesity related parameters headache frequency and their association with headache frequency

Parameter	Baseline (Mean ± SD)	Follow-up (Mean ± SD)	p-value† (within-subject)	Headache frequency(Mean ± SD)	Follow up increase headache (Mean ± SD)	p-value‡ (between-follow up group)
Weight	74.66±18.94	75.35 ± 18.96	0.314	77.01 ± 20.81	73.37.35 ± 16.69	0.192
BMI*	29.41 ± 7.22	26.46 ± 7.74	0.871	30.78 ± 8.21	28.08±6.98	0.018
Waist	94.69±15.84	95.11 ± 16.23	0.465	96.54 ± 17.86	93.58±14.22	0.214

† Paired t-test (baseline vs follow-up within subjects)

‡ Independent t-test (between participants with vs. without increased headache frequency)

* BMI=Body mass index

SD=standard deviation

**Table 4:** Association of Obesity Type with Headache Frequency(HF) at Baseline, Follow-up, and Change

Group	n (%)	Baseline HF (Mean± SD)	Follow-up HF (Mean ± SD)	p-value† (HF baseline vs follow-up)	% With Frequency Increase	p-value‡ (increase %)
Normal weight	105(56.45)	6.40 ± 5.82	7.50 ± 56.92		64.4%	
Generalized obesity only	07(3.76)	7.00 ± 5.35	8.43 ± 6,29	p = 0.947	2.2%%	0.086
Both generalized and central obesity	74(39.78)	6.26 ± 6.06	7.66 ± 6.96		33.3%	

† ANOVA for headache frequency across groups

‡ Chi-square for comparing % with increased frequency

SD=standard deviation

**Associations**: Univariate analysis linked known triggers (p=0.020), headache frequency (p=0.010), follow-up increases in frequency/severity (p=0.040), poor diet (p<0.001), medication non-compliance (p=0.030), high BMI (p=0.030), high WHtR (p=0.030), and follow-up weight/WC increases (p<0.010) to increased headache burden. However, multivariable logistic regression revealed only known triggers (OR 2.04, 95% CI 1.02–4.05; p=0.040) as significant predictors; follow-up increases in weight and WC were marginal (OR 1.94, 95% CI 0.97–3.89; p=0.050). Obesity measures and central obesity were not independently predictive. Notably, poor dietary habits (OR 0.26, p<0.001) and non-compliance (OR 0.43, p=0.010) were significantly associated with increased headache burden (Table 5).

**Table 5:** Univariate and multivariate binary logistic regression analysis for association of patient's clinical and obesity related parameters with increased frequency and severity of migraine attacks

Variable	Univariate analysis Odds ratio (95% CI)	p-value	Multivariate analysis Odds ratio (95% CI)	p-value
Age	1.00(0.98-1.03)	0.420	1.02(0.99-1.05)	0.08
Male	0.61 (0.30-1.27)	0.19	0.60(0.26-1.309)	0.23
Episodic migraine	0.32 (0.15-0.99)	0.04	0.34(0.11-1.03)	0.05
Known triggers	1.34 (0.75-2.39)	0.31	2.04(1.02-4.05)	0.04
Poor dietary habits	1.98 (1.39-2.82)	<0.001	0.26(0.13-0.52)	<0.001
Non-compliant to medications	0.52(0.29-0.94)	0.03	0.43(0.22-0.85)	0.01
High BMI	1.35(1.02-1.78)	0.03	1.56(0.54-4.53)	0.40
High waist	1.71(0.92-3.17)	0.08	1.49(0.64-3.46)	0.35
High waist height ratio	1.91(1.06-3.47)	0.03	1.05(0.35-3.13)	0.92
High FU body weight and waist	2.30(1.26-4.20)	<0.001	1.94(0.97-3.89)	0.05

Hosmer-lemeshow goodness-of-fit test for logistic regression analysis: 0.87; .BMI=Body mass index; Fu= Followup

Overall, while obesity is prevalent among migraineurs, its influence on headache burden appears to be mediated primarily through lifestyle and behavioral factors rather than obesity per se.

## Discussion

This study explored the influence of obesity on migraine frequency and severity, with a predominantly female cohort (79%), reflecting the known higher prevalence of migraine among women, particularly during reproductive years (11-15, 16). Estrogen has been implicated in this sex disparity, contributing to the higher migraine burden in females (17). In our sample, elevated BMI, WC, and WHtR were found in 43.1%, 67.2%, and 41.4% of patients, respectively—consistent with existing literature reporting both generalized and central obesity among migraineurs, particularly in reproductive-aged women. (3-4-7-8 and 18) Nauman et al. similarly reported high BMI in 45.4% of male and 39.3% of female migraineurs, with elevated WHtR in 81.8% and 64.3%, respectively (9).

The mean age of participants was 40.64 ± 13.21 years, with a mean diagnosis duration of 8.38 ± 7.00 years. A significant proportion were over 40 and had comorbidities, which is notable given the established co-occurrence of migraine with various chronic conditions—many of which, like obesity, are interconnected through shared genetic and environmental risk factors (2-19, 20). Comorbidities such as hypertension (HTN), depression, endocrine, psychiatric, epileptic, cardiovascular, cerebrovascular, and autoimmune disorders are frequently documented in migraine patients (21).

Our study found elevated systolic BP and BMI in migraineurs with comorbidities. Prior studies have reported higher diastolic BP in female migraineurs compared to non-migraineurs, (22), and an increased risk of HTN (by 9% in those with aura and 21% in those without aura) (23). While most comorbidities have been linked to chronic migraine, (21) our predominantly episodic migraine sample still exhibited significant comorbidity rates, aligning with literature that indicates comorbidities in episodic migraine may increase the risk of progression to chronic migraine (24). Moreover, depression and other comorbid conditions are known risk factors for increased headache frequency and chronicity (2).

Interestingly, high-frequency headaches were more common among migraineurs without comorbidities in this study. Over a 12-month follow-up, weight gain was observed in 41.4% of participants—more so among those with comorbidities—reinforcing findings that link migraine to metabolic and behavioral dysregulation (2). Additionally, 51.6% of patients reported increased headache frequency and intensity, regardless of comorbidity status. This aligns with other studies and meta-analyses indicating that obesity is associated with increased headache frequency and greater risk for chronic migraine in overweight individuals (2-21, 25).

Several treatment and lifestyle-related factors are associated with worsening headache frequency in episodic migraine patients, potentially triggering chronicity (8-26, 27). Similarly, we observed that high-frequency headaches and increased frequency at follow-up were most prevalent among episodic migraineurs, highlighting their vulnerability to progression.

Patients reporting known headache triggers were more likely to experience severe attacks—a finding supported by literature linking triggers to increased attack frequency and intensity (28). Central sensitization, involving hyperexcitability of second-order neurons in the trigemino-cervical complex, likely plays a role by lowering the threshold for nociceptive activation (29).

Our study also observed that migraineurs with poor dietary habits, poor adherence to preventive therapy, high BMI, elevated WHtR, and increased weight and WC at follow-up experienced greater headache frequency and severity. Obesity, unhealthy lifestyle factors, and poor diet are all recognized as major contributors to reduced quality of life in chronic conditions (30). Various studies have reported associations between specific dietary patterns and migraine: for instance, fried and fast food consumption has been linked to increased migraine attacks, while high intake of fruits, vegetables, legumes, and fish may reduce headache frequency (31-32, 33).

While BMI has been widely linked to migraine frequency. (25-34) our study did not find independent associations between baseline BMI or WHtR and increased headache burden in multivariate analysis. However, increases in weight and WC at follow-up approached statistical significance (p=0.05), suggesting a potential cumulative or mediating role of obesity over time. The absence of significant within-subject changes over time suggests short-term weight changes may not directly affect headache patterns. Nonetheless, the observed association between higher follow-up BMI and increased headache frequency supports the possibility of an underlying link between weight gain and migraine progression.

Patients with both generalized and central obesity showed a consistent trend toward higher headache frequency at both baseline and follow-up, although this was not statistically significant. These trends align with literature suggesting that central adiposity may play a more important role than generalized obesity in migraine chronicity (8-9). Future studies with larger samples and extended follow-up periods are needed to further investigate these associations and their potential clinical implications.

Importantly, this study underscores that obesity should not be evaluated in isolation when assessing migraine burden. Changes in anthropometric parameters must be considered within the broader context of behavioral patterns, treatment adherence, and trigger exposure.

This study had several limitations. First, the use of convenience sampling and a relatively small sample size may limit generalizability. Second, although the study followed patients for 12 months, this duration may not be sufficient to observe meaningful long-term changes in body weight and migraine progression. Third, it did not evaluate the relationship between anthropometric indices and headache duration or functional disability. Moreover, anthropometric and blood pressure measurements were performed by a single trained nurse, and only one reading was recorded, introducing potential measurement bias.

In conclusion, obesity-related factors, particularly elevated BMI and WC, were common among migraineurs in this study but did not independently predict increased headache frequency or severity after adjusting for behavioral and lifestyle variables. Instead, poor dietary habits, medication non-compliance, and known headache triggers emerged as significant contributors to headache burden. These findings underscore the need for personalized, comprehensive migraine management strategies that address lifestyle modification, trigger avoidance, and adherence to treatment. While weight monitoring remains important, especially in patients with poor dietary practices or low compliance, obesity appears to exert its effect primarily through behavioral mediators rather than direct mechanisms. Future longitudinal and mechanistic studies are warranted to better understand how central obesity and inflammation influence migraine chronicity and related outcomes.
